# Hupresin Retains Binding Capacity for Butyrylcholinesterase and Acetylcholinesterase after Sanitation with Sodium Hydroxide

**DOI:** 10.3389/fphar.2017.00713

**Published:** 2017-10-10

**Authors:** Seda Onder, Emilie David, Ozden Tacal, Lawrence M. Schopfer, Oksana Lockridge

**Affiliations:** ^1^Department of Biochemistry, School of Pharmacy, Hacettepe University, Ankara, Turkey; ^2^Eppley Institute, University of Nebraska Medical Center, Omaha, NE, United States; ^3^CHEMFORASE, Mont-Saint-Aignan, France

**Keywords:** Hupresin affinity gel, no-ghost erythrocyte AChE, immobilized monoclonal antibodies, mass spectrometry, butyrylcholinesterase

## Abstract

Hupresin is a new affinity resin that binds butyrylcholinesterase (BChE) in human plasma and acetylcholinesterase (AChE) solubilized from red blood cells (RBC). Hupresin is available from the CHEMFORASE company. BChE in human plasma binds to Hupresin and is released with 0.1 M trimethylammonium bromide (TMA) with full activity and 10–15% purity. BChE immunopurified from plasma by binding to immobilized monoclonal beads has fewer contaminating proteins than the one-step Hupresin-purified BChE. However, when affinity chromatography on Hupresin follows ion exchange chromatography at pH 4.5, BChE is 99% pure. The membrane bound AChE, solubilized from human RBC with 0.6% Triton X-100, binds to Hupresin and remains bound during washing with sodium chloride. Human AChE is not released in significant quantities with non-denaturing solvents, but is recovered in 1% trifluoroacetic acid. The denatured, partially purified AChE is useful for detecting exposure to nerve agents by mass spectrometry. Our goal was to determine whether Hupresin retains binding capacity for BChE and AChE after Hupresin is washed with 0.1 M NaOH. A 2 mL column of Hupresin equilibrated in 20 mM TrisCl pH 7.5 was used in seven consecutive trials to measure binding and recovery of BChE from 100 mL human plasma. Between each trial the Hupresin was washed with 10 column volumes of 0.1 M sodium hydroxide. A similar trial was conducted with red blood cell AChE in 0.6% Triton X-100. It was found that the binding capacity for BChE and AChE was unaffected by washing Hupresin with 0.1 M sodium hydroxide. Hupresin could be washed with sodium hydroxide at least seven times without losing binding capacity.

## Introduction

Acetylcholinesterase (AChE) and butyrylcholinesterase (BChE) are present in human blood and in most other tissues (Manoharan et al., 2007). AChE has an important function in cholinergic nerve impulse transmission. BChE hydrolyzes the hunger hormone, octanoyl-ghrelin to inactive products and thus has a role in development of obesity (Chen et al., 2016, 2017). Both enzymes are inhibited by nerve agents and organophosphorus pesticides. Human BChE is an excellent bioscavenger of nerve agents. Animals pretreated with pure human BChE are completely protected from the toxicity of nerve agents at doses of nerve agent that are lethal to untreated animals (Broomfield et al., 1991; Raveh et al., 1997; Mumford et al., 2010).

Human AChE is bound to the membrane of red blood cells (RBC) through a glycophospholipid anchor, where it is a disulfide linked dimer of 130 kDa (Rosenberry and Scoggin, 1984; Toutant et al., 1991). BChE in plasma and serum is a sugar-coated tetramer of four identical subunits with a molecular weight of 340 kDa. 1 mL of whole blood contains 0.5 μg of AChE bound to RBC and up to 5 μg of BChE in plasma (Bartels et al., 2000). Plasma also contains 0.008 μg/mL of soluble AChE (Brimijoin and Hammond, 1988).

Exposure to nerve agents and organophosphorus pesticides can be monitored by liquid chromatography-tandem mass spectrometry (LC-MS/MS) of pepsin-digested immunopurified BChE (Fidder et al., 2002; Pantazides et al., 2014; Mathews et al., 2017). A method for immunopurifying and analyzing RBC AChE by LC-MS/MS has also been developed (Dafferner et al., 2017). A new affinity gel, Hupresin, has been proposed as an alternative to immunopurification of AChE and BChE from blood. Large scale purification of BChE from frozen Cohn fraction IV-4 is successfully achieved by using Hupresin as the second chromatography step following ion exchange chromatography. A sanitation step with 0.1 M sodium hydroxide removes contaminants that are not released with sodium chloride. The purpose of the present work was to determine the stability of Hupresin to multiple washings with 0.1 M sodium hydroxide after repeated chromatography of plasma BChE or solubilized RBC AChE.

## Materials and methods

Emilie David at the CHEMFORASE Company, Mont-Saint-Aignan, France, synthesized the ligand and crosslinked it to a Sepharose chromatographic support. The name of the affinity gel is Hupresin (emilie.david@chemforase.com). Volunteer donor blood was obtained from the University of Nebraska Hospital Blood Bank. Pure human BChE was purified from Cohn fraction IV-4 by chromatography on Q-ceramic ion exchanger followed by chromatography on Hupresin affinity gel. Dextran sulfate (Sigma D-6001, MW 500,000) and standard chemicals were from Sigma.

### Enzyme activity assays

AChE activity was measured in 0.1 M potassium phosphate pH 7.0 at 25°C with 1 mM acetylthiocholine iodide in the presence of 0.5 mM 5,5″-dithiobis(2-nitrobenzoic acid) on a Gilford spectrophotometer interfaced to a MacLab data recorder (ADInstruments, Inc.). No-ghost RBC AChE solutions (5 μL) were preincubated with 1.98 mL of 0.5 mM 5,5″-dithiobis(2-nitrobenzoic acid) in buffer for 10 min or more to deplete free sulfhydryl groups before the AChE reaction with acetylthiocholine was started by addition of 0.02 mL of 0.1 M acetylthiocholine iodide. The increase in absorbance at 412 nm was converted to micromoles acetylthiocholine hydrolyzed using the extinction coefficient 13,600 M^−1^ cm^−1^ (Ellman et al., 1961). Units of activity are expressed as micromoles per min. AChE units/mL (pH 7) were converted to mg/mL using the conversion factor of 5,000 units/mg (Rosenberry and Scoggin, 1984).

BChE activity was measured in 0.1 M potassium phosphate pH 7.0 at 25°C with 1 mM butyrylthiocholine iodide as above. Units of activity are expressed as micromoles per min. BChE units/mL were converted to mg/mL using the conversion factor of 720 units/mg.

### Binding of plasma BChE to hupresin

Hupresin (2 mL) in Pharmacia column C10/10 was equilibrated with 20 mM TrisCl pH 7.5, 0.05% azide. Human plasma (100 mL) with an activity of 2 u/mL was pumped onto the 2 mL Hupresin at room temperature at a flow rate of 0.2 mL per min using Pharmacia pump P1 with 2.1 mm tubing. The first five runs used plasma collected in citrate phosphate dextrose anticoagulant. The 6 and 7th runs used plasma delipidated with dextran sulfate and 50 mM calcium chloride. To avoid precipitating calcium phosphate, the buffer for chromatography of plasma was 20 mM TrisCl pH 7.5, 0.05% azide. After the plasma had been loaded, Hupresin was washed with 8 mL of Tris buffer and 22 mL of 0.3 M NaCl in Tris buffer. BChE was eluted with 10 mL of 0.1 M trimethylammonium bromide (TMA) in pH 7.5 TrisCl buffer. Hupresin was washed with 2 M NaCl and desalted with water before it was cleaned with 0.1 M NaOH, and equilibrated for the next application of 100 mL plasma.

### No-ghost method for solubilizing RBC AChE

Frozen human RBC (200 mL) were thawed and diluted with 300 mL of 1% Triton X-100 in PBS, 0.1% azide to solubilize membrane-bound AChE (final Triton concentration was 0.6%). The insoluble debris was removed by centrifugation at 10°C in a Sorvall RC 5C plus centrifuge, using an SS34 rotor, for 30 min, at 14,000 rpm (23,400 × g). It was essential to remove the debris to avoid clogging the chromatography gel. The solubilized no-ghost RBC AChE solution was very red, but not viscous. No-ghost RBC AChE activity was 1.6 u/mL. The solubilized RBC AChE preparation was named no-ghost RBC AChE to distinguish it from the standard protocol for extracting AChE from the red blood cell membrane in which cells are lysed and washed to remove hemoglobin, leaving a white pellet of red cell ghosts (Dodge et al., 1963).

### Binding of no-ghost RBC AChE to hupresin

A 30 mL aliquot of no-ghost RBC AChE in 0.6% Triton X-100 with an activity of 1.6 u/mL was loaded on 2 mL Hupresin packed in a Pharmacia C10/10 column. The column was washed with 20 mL of 0.1 M potassium phosphate pH 7.5, 0.05% azide, 20 mL of 1 M NaCl in buffer, 20 mL of 3 M NaCl in buffer, and 20 mL water. We have not found a solvent that elutes AChE from Hupresin quantitatively while retaining AChE activity. However, this is not a problem for experiments that require denatured AChE. Denatured AChE was eluted from Hupresin with 1% trifluoroacetic acid. The Hupresin was cleaned before application of the next 30 mL aliquot of no-ghost RBC AChE by pumping 20 mL of 0.1 M NaOH for 45 min, followed by neutralization and equilibration with 0.1 M potassium phosphate pH 7.5, 0.05% azide.

### Recycling hupresin with 0.1 M NaOH

Hupresin (2 mL) packed in a C10/10 Pharmacia column was cleaned by pumping 20 mL of 0.1 M NaOH for 45 min, followed by neutralization and equilibration with 20 mM TrisCl pH 7.5, 0.05% azide or with 0.1 M potassium phosphate pH 7.5, 0.05% azide.

### Delipidation of fatty plasma

Fatty plasma was identified on the basis of its cloudy appearance and the presence of a layer of fat on top of the plasma after centrifugation at 4°C. Fatty plasma was delipidated by the method of Masseyeff et al. (1965). A 10% dextran sulfate solution was prepared in water. A 1 mL aliquot of 10% dextran sulfate was slowly added to 100 mL of fatty plasma with mixing, followed by 2 mL of 2.5 M CaCl_2_ to make 50 mM calcium chloride. Centrifugation in a Sorvall RC%C Plus centrifuge, using an SS34 rotor, at 15,000 rpm (27,000 × g), for 30 min, at 10°C yielded a clear plasma solution free of visible fat.

### SDS gel electrophoresis

Two types of slab gels were used—precast 4–20% gradient gels from BioRad (no stacking gel) and homemade 4–30% gradient gels with a 4% stacking gel cast in a Hoefer SE600 system. Gels were stained with Coomassie blue. Proteins in the Coomassie blue stained bands were identified by LC-MS/MS after the proteins were extracted from gel slices and digested with trypsin, as described (Peeples et al., 2005).

### Non-denaturing gel stained for BChE activity

Slab gels, 4–30% gradient, poured in an SE600 Hoefer apparatus were electrophoresed for 24 h at 320 volts constant voltage at 4°C before they were stained for BChE activity by the method of Karnovsky and Roots using butyrylthiocholine iodide as substrate (Karnovsky and Roots, 1964).

### Immunopurification of BChE from plasma

Monoclonal antibodies B2 18-5 (accession KT189143 heavy chain, KT189144 light chain) and mAb2 (accession KJ141199 heavy chain, KJ141200 light chain) were covalently bound to CNBr-activated Sepharose 4B Fast Flow (Peng et al., 2015). A 20 μL aliquot of beads containing 33 μg of bound antibody was incubated with 1.5 mL human plasma overnight at room temperature on a rotating platform. Beads were washed with PBS on a 0.45 micron filter until the absorbance at 280 nm of the flow through was <0.04. The beads were desalted by washing with water. Bound proteins were eluted with 2 × 50 μl of 1% trifluoroacetic acid (TFA). The TFA extract was dried, dissolved in reducing SDS gel loading buffer, boiled 3 min and loaded on an SDS gel.

### Liquid chromatography tandem mass spectrometry

Proteins that eluted off Hupresin along with BChE or RBC AChE, as well as proteins from SDS gel bands were identified by liquid chromatography tandem mass spectrometry (LC-MS/MS). Data acquisition was performed with a Triple-TOF 6,600 mass spectrometer (ABI Sciex, Framingham, MA) fitted with a Nanospray III source (AB Sciex, Framingham, MA) and a Pico Tip emitter (# FS360-20-10-N-5-C12, New Objectives, Woburn, MA). The ion spray voltage was 2,700 V, declustering potential 60 V, curtain gas 30 psi, nebulizer gas 10 psi, and interface heater temperature 150°C.

Peptides were introduced into the mass spectrometer using ultra high pressure liquid chromatography. A splitless Ultra 1D Plus ultra-high pressure chromatography system (Eksigent, Dublin, CA) was coupled to the Triple-TOF via a cHiPLC Nanoflex microchip column system (Eksigent, Dublin, CA). The Nanoflex system uses a replaceable microfluidic trap column and a replaceable separation column. Both are packed with ChromXP C_18_ (3 μm, 120 Å particles; Trap: 200 μm × 0.5 mm; Separation: 75 μm × 15 cm). Chromatography solvents were water/acetonitrile/formic acid (A: 100/0/0.1%, B: 0/100/0.1%). Picomole amounts of sample, in a 5 μl volume, were loaded. Trapping and desalting were carried out at 2 μL/min for 15 min with 100% mobile phase A. Separation was obtained with a linear gradient 5%A/95%B to 70%A/30% B over 60 min at a flow rate of 0.3 μL/min.

Peptides present in the data were identified by matching to the Swiss Prot or National Center for Biotechnology Information (NCBI) non-redundant databases, and corresponding proteins were identified. The Paragon algorithm in Protein Pilot v 5.0 (AB Sciex) was used to search the databases. Database search parameters specified the protease used for digestion, the state of cysteine alkylation, the species from which the sample was derived, the “ID focus” which was typically “Biological modifications,” the database, and the type of “Search effort” which was typically “Thorough.” Protein Pilot software was used to inspect the database search results.

### MALDI-TOF mass spectrometry

The goal was to identify a brown substance that co-eluted with RBC AChE from Hupresin in 1% TFA. A 1 μL aliquot was applied to a MALDI plate with 2,5-dihydroxybenzoic acid (DHB) matrix. Spectra were acquired in positive mode using a laser voltage of 5,000 on a 4,800 MALDI-TOF/TOF mass spectrometer (AB Sciex).

## Results

### Hupresin

The affinity ligand in Hupresin is a custom synthesized hybrid of tacrine and huperzine (Ronco et al., 2011; Brazzolotto et al., 2012). Emilie David at the CHEMFORASE company, Mont-Saint-Aignan, synthesized the ligand and crosslinked it to the 10 atom spacer of ECH-Sepharose 4B (GE Healthcare 17-0571-01). The name of the affinity gel is Hupresin.

### Binding capacity of hupresin for BChE

Human plasma has a significantly lower BChE activity than serum because plasma collected by the American Red Cross from volunteer donors is diluted with liquid anticoagulant. In contrast serum is undiluted. Plasma samples have an average BChE activity of 2 units/mL. This is 270 μg BChE in 100 mL plasma, calculated using a specific activity of 720 units/mg for pure HuBChE. 2 mL of Hupresin bound 80–90% of the BChE in 100 mL plasma as indicated in Table 1. Percent bound in Table 1 was the amount of BChE activity bound to Hupresin after the activity in the flow through, buffer wash, and 0.3 M NaCl was subtracted. The binding capacity of Hupresin for BChE in plasma is about 0.1 mg per mL Hupresin. When BChE is partially purified by ion exchange chromatography before it is loaded on Hupresin the binding capacity increases at least 3-fold to 0.3 mg/mL Hupresin.

**Table 1:** Purification of BChE from 100 mL plasma in seven consecutive trials on the same 2 mL Hupresin.

**Trial**	**% BChE bound**	**Units/mg**	**Fold purified**	**0.1 M NaOH wash**
1	85	62.5	2,041	1
2	82	130	2,256	2
3	80	112	1,937	3
4	87	105	1,822	4
5	90	105	1,816	5
		Av 103 ± 22	Av 1974 ± 163	
6	77^a^	106	1,513	6
7	79^a^	102	1,454	7

*Hupresin was washed with 0.1 M NaOH before each trial*.

a *Plasma in trials 6 and 7 had been delipidated with dextran sulfate and CaCl_2_. The lower amount of BChE bound in trials 6 and 7 is due to interference by Dextran sulfate in the delipidated plasma*.

### BChE binding capacity is undiminished by washing hupresin with 0.1 M NaOH

Hupresin was washed with 0.1 M NaOH, neutralized, and equilibrated with 20 mM TrisCl pH 7.5 before chromatographing 100 mL plasma, including before the first chromatography. Table 1 shows that the % BChE bound in trial 5 was no lower than in trial 1. The lower amount of BChE bound in trials 6 and 7 is due to interference by Dextran sulfate in the delipidated plasma. Though Dextran sulfate had been removed by centrifugation so that the delipidated plasma appeared clear, a visible layer of Dextran particles accumulated on top of the Hupresin column while 100 mL of delipidated plasma was pumped onto the column. Residual dextran particles were removed from delipidated plasma by filtration through a 0.8 μ filter. The filtered, delipidated plasma left no dextran particles on the Hupresin when 100 mL was pumped onto 2 mL Hupresin. It was concluded that Hupresin washed seven times with 0.1 M NaOH retained its binding capacity for BChE in plasma.

### Purity of BChE recovered from passage of plasma over hupresin

Serum contains about 60 mg/mL protein, the most abundant being albumin at 40 mg/mL and immunoglobulin at 16 mg/mL (Fahey and McKelvey, 1965; Peters, 1996). Plasma diluted with anticoagulant contains about 40 mg/mL protein. The specific activity of BChE in plasma is two units in 40 mg or 0.05 u/mg. Pure BChE has a specific activity of 720 u/mg. Dividing 720 u/mg by 0.05 u/mg yields 14,400 which means BChE has to be purified 14,400-fold to obtain pure BChE from plasma. A single passage over Hupresin did not achieve this level of purity. Table 1 shows that BChE was purified about 2,000-fold by affinity chromatography on Hupresin. This corresponds to 10–15% pure BChE. A 2-fold purer BChE with a specific activity of 217 u/mg was obtained by collecting ten 1 mL fractions rather than a single 10 mL fraction during elution with 0.1 M TMA. In this experiment the purest fractions were #4 (217 u/mg), #5 (150 u/mg) and #6 (130 u/mg).

**Figure 1:**
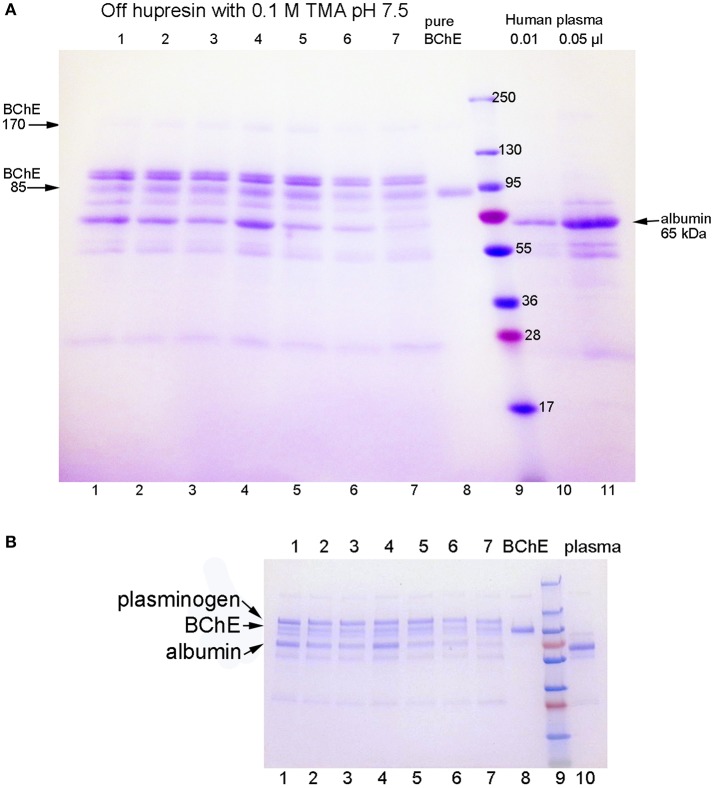
SDS gels stained with Coomassie blue. Gel **A** is a 4–30% gradient gel, 14 × 11 cm, with a 4% stacking gel (4 cm). Gel **B** is a 4–20% gradient precast gel (8 × 6 cm) without a stacking gel. Identical samples were run on the 2 gels. Lanes 1–7: partially purified BChE from 100 mL plasma that was eluted from 2 mL Hupresin with 0.1 M TMA pH 7.5, in seven consecutive trials. The Hupresin was washed with 0.1 M sodium hydroxide before each trial. Lane 8: pure human BChE. Lanes 10 and 11: human plasma.

The SDS gels in Figure 1 are for BChE samples eluted from Hupresin in seven consecutive trials for 100 mL plasma applied to 2 mL Hupresin. The SDS gel in Figure 1A was prepared in-house with a 4–30% polyacrylamide gradient separating gel and a 4% stacking gel. Bands for BChE are present at 85 and 170 kDa. Major contaminating bands are at 100 and 65 kDa. The same samples were run on a precast 4–20% gradient gel and no stacking gel in Figure 1B. Comparison of the two gels shows that proteins in lanes 1–8 migrate slower on the precast gel, relative to the molecular weight markers, than on the in-house gel. The result is that apparent molecular weights determined from the precast gel appear to be larger. Thus, the molecular weight of BChE in Figure 1A is 85 kDa but appears to be 100 kDa in Figure 1B. The correct mass for the glycosylated BChE monomer is 85 kDa. In-gel digestion of bands in Figure 1B followed by mass spectrometry analysis identified the major contaminants as plasminogen at 100 kDa and albumin at 65 kDa. Weaker contaminant bands at 55 and 25 kDa are probably the heavy and light chains from immunoglobulin.

The sample in lanes 10 and 11 of Figure 1A is the starting plasma. It shows the position of albumin on the gel. Immunoglobulin heavy and light chains are at 55 and 25 kDa. The plasma sample has no band for BChE because the concentration of BChE in plasma is 10,000-fold lower than the concentration of albumin.

Table 2 lists the proteins identified by mass spectrometry in the 15% pure BChE sample eluted off Hupresin. The most abundant protein as estimated from peptide counts was BChE. Albumin and plasminogen were confirmed as abundant contaminants. 11 additional proteins are listed in Table 2 as contaminants in the preparation. Protein contaminants with a peptide count lower than that of fibronectin were excluded from Table 2.

**Table 2:** Proteins identified in a 15% pure BChE sample recovered after affinity chromatography of plasma on Hupresin.

**Accession number**	**Name**	**Peptide counts**	**MW (UniProt)^a^**	**Gene**
P06276	Butyrylcholinesterase	189	68,418	BCHE
P02768	Albumin	149	69,367	ALB
P00747	Plasminogen	110	90,569	PLG
P01024	Complement 3	73	187,148	C3
P01023	Alpha-2-macroglobulin	59	163,291	A2M
P04114	Apolipoprotein B-100	47	515,605	APOB
P0C0L5	Complement C4-B	41	192,751	C4B
P02675	Fibrinogen beta chain	41	55,928	FGB
P0DOX5	Immunoglobulin gamma-1 heavy chain	40	49,330	
P0C0L4	Complement C4-A	40	192,785	C4A
P02671	Fibrinogen alpha chain	37	94,973	FGA
P02787	Serotransferrin	37	77,064	TF
P01009	Alpha-1-antitrypsin	32	46,737	SERPINA1
P02751	Fibronectin	32	262,625	FN1

a *Protein molecular weights (MW) are taken from the UniProt database which includes the signal peptide, but excludes glycans and other posttranslational modifications. The molecular weight of the BChE monomer is actually 85 kDa when the 28 amino acid signal peptide is subtracted and 9 asparagine-linked glycans are added*.

### One step purification of BChE from plasma using hupresin or antibody beads

**Figure 2:**
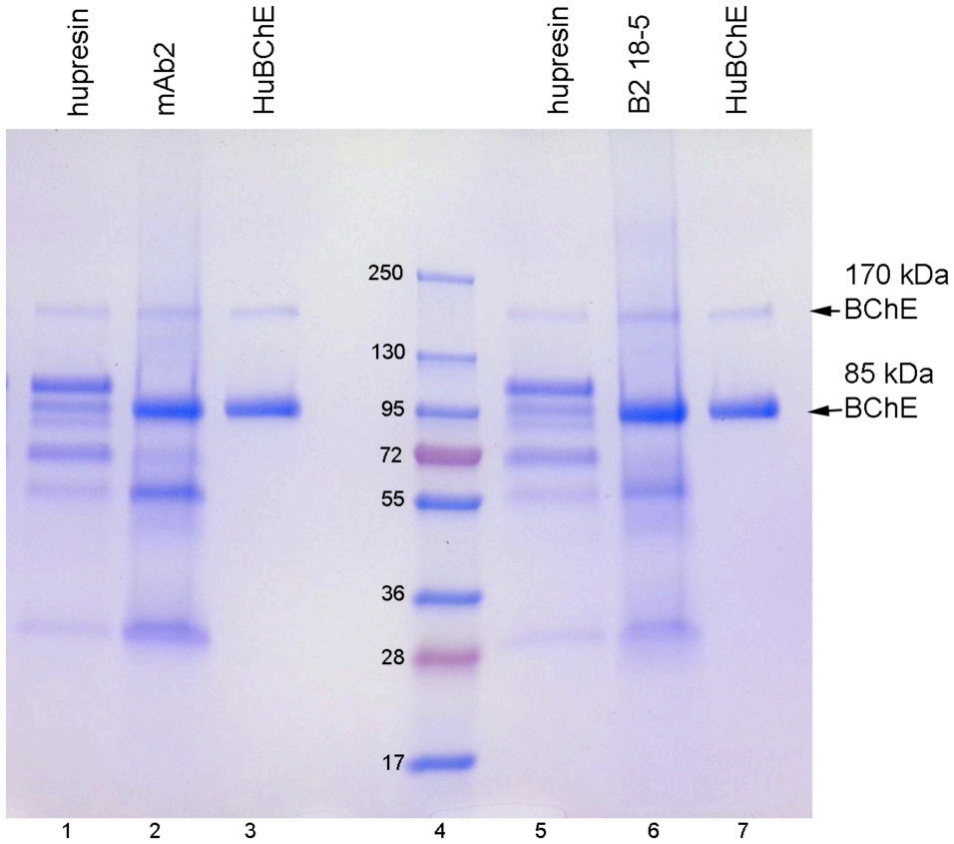
Comparison of BChE purity following one-step purification on Hupresin or on immobilized monoclonal beads. The precast 4–20% gradient SDS gel was stained with Coomassie blue. Lanes 1 and 5, BChE eluted from Hupresin with 0.1 M TMA pH 7.5. Lane 2, BChE eluted from monoclonal mAb2 with 1% TFA. Lanes 3 and 7, pure human BChE. Lane 6, BChE eluted from monoclonal B2 18-5 with 1% TFA. Lane 4, molecular weight markers.

When choosing a one-step method for extracting BChE from plasma, the best options are affinity chromatography on Hupresin or immunopurification on immobilized antibody beads. Figure 2 compares BChE recovered from one-step chromatography on Hupresin to BChE recovered from binding to monoclonal antibodies mAb2 and B2 18-5. The immunopurified BChE is purer. The principal contaminants are at about 55 and 25 kDa and probably arise from the antibodies used for purification. It is acknowledged that the immunopurified BChE has no activity because it must be dissociated from the antibody with a denaturant, 1% TFA.

Table 3 lists the proteins identified by mass spectrometry in the plasma sample eluted from immobilized antibody B2 18-5 with 1% trifluoroacetic acid. Essentially identical results were obtained when immobilized antibody mAb2 was used. BChE is the most abundant protein in Table 3. Comparison of Tables 2, 3 shows there are far fewer contaminating proteins in the immunopurified BChE than in the one-step Hupresin purified BChE. Contaminating proteins identified by LC-MS/MS range in molecular weight from 25 to 515 kDa for the two preparations. Only the most abundant proteins have corresponding bands on the Coomassie blue stained gels in Figures 1, 2.

**Table 3:** Proteins identified in immunopurified BChE eluted from B2 18-5 beads with acid.

**Accession number**	**Name**	**Peptide counts**	**MW (UniProt)**	**Gene**
P06276	Butyrylcholinesterase	136	68,418	BCHE
–	Immunoglobulins	93	55,000, 25,000	–
P02751	Fibronectin 1	29	262,625	FN1
Q15485	Ficolin-2-isoform b	5	34,001	FCN2
P19827	Inter-alpha-trypsin inhibitor	5	101,389	ITIH1

### Minor BChE isozymes

**Figure 3:**
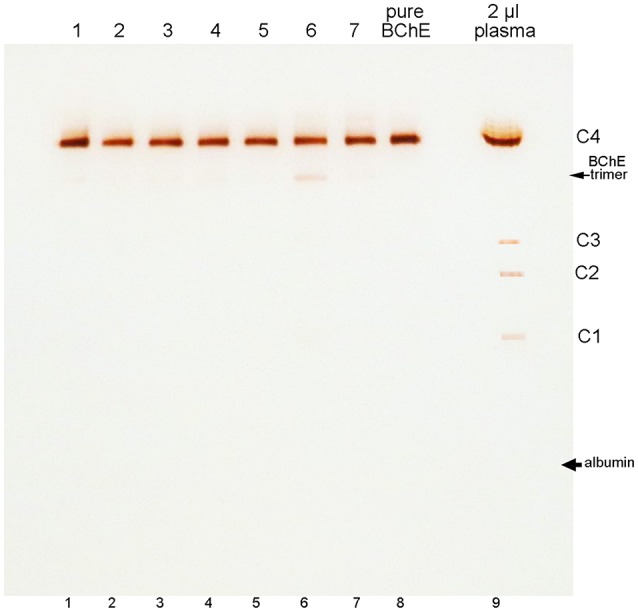
Nondenaturing 4–30% gradient gel stained for BChE activity. Samples in lanes 1–5 are for 100 mL plasma samples chromatographed repeatedly on 2 mL Hupresin. The Hupresin was sanitized with 0.1 M NaOH between each chromatography. Samples in lanes 6 and 7 are delipidated plasma samples chromatographed on Hupresin. The Hupresin was sanitized with 0.1 M NaOH before and after each chromatography. Lane 8 contains pure BChE obtained by a combination of ion exchange and Hupresin chromatography. BChE in plasma (lane 9) consists predominantly of tetramers designated C4. The minor C1 and C3 isozymes of BChE are monomers and dimers of BChE. The C2 isozyme is a conjugate of BChE and albumin (Masson, 1989).

BChE in plasma consists predominantly of tetramers. On non-denaturing gel electrophoresis bands for BChE monomers, dimers, and trimers are also seen, though their abundance is low (refer to the legend of Figure 3 for band annotation). Figure 3 shows that the minor BChE bands found in plasma are missing from BChE samples that have been chromatographed on Hupresin (lanes 1–7). Delipidated plasma in lane 6 developed a new band at the position of trimeric BChE.

The position of albumin is indicated in Figure 3. It was identified by counterstaining the gel with Coomassie blue. Albumin (65 kDa) separates from BChE (340 kDa) on a non-denaturing gel, but the proteins run close to each other on a reducing SDS gel where the predominant BChE mass is 85 kDa.

### Recovery of RBC AChE from 2 mL hupresin

AChE binds very tightly to Hupresin. Triton X-100 and hemoglobin in the solubilized no-ghost RBC preparations did not interfere with AChE binding to Hupresin. Hemoglobin eluted from Hupresin without binding; the flow through was as red as the starting material. Only 10–20% of the AChE activity eluted from Hupresin during the loading and washing steps.

The effect of cleaning the Hupresin with 0.1 M NaOH on AChE binding was tested. After purification of AChE from a 30 ml aliquot of no-ghost RBC, 20 mL of 0.1 M NaOH was pumped over the 2 mL of Hupresin for 45 min, followed by neutralization and equilibration with 0.1 M potassium phosphate pH 7.5, 0.05% azide. The cycle was repeated seven times. A similar quantity of no-ghost RBC AChE bound to Hupresin after the 7th wash as after the first. It was concluded that Hupresin retained its binding capacity for RBC AChE after being washed with 0.1 M NaOH at least seven times.

No solvent was identified that quantitatively eluted native, active AChE from Hupresin. Three molar NaCl was routinely used to separate contaminating proteins from AChE without dislodging AChE. Hupresin affinity chromatography is not suitable for purifying RBC AChE in a native form having AChE activity. However, the tight binding of Hupresin to AChE can be used to advantage when the experiment requires purified AChE protein, but does not require AChE activity. For example, denatured AChE recovered from RBC can be used for mass spectrometry analysis of nerve agent and organophosphorus toxicant exposure.

After the 2 mL Hupresin column was washed with 3 M NaCl, the Hupresin was light brown in color. Some of the brown color co-eluted with AChE in 1% TFA. The Hupresin returned to its original white color after it was washed with 0.1 M NaOH. MALDI-TOF mass spectrometry analysis identified the brown material as heme, with a mass of 616 Da.

**Figure 4:**
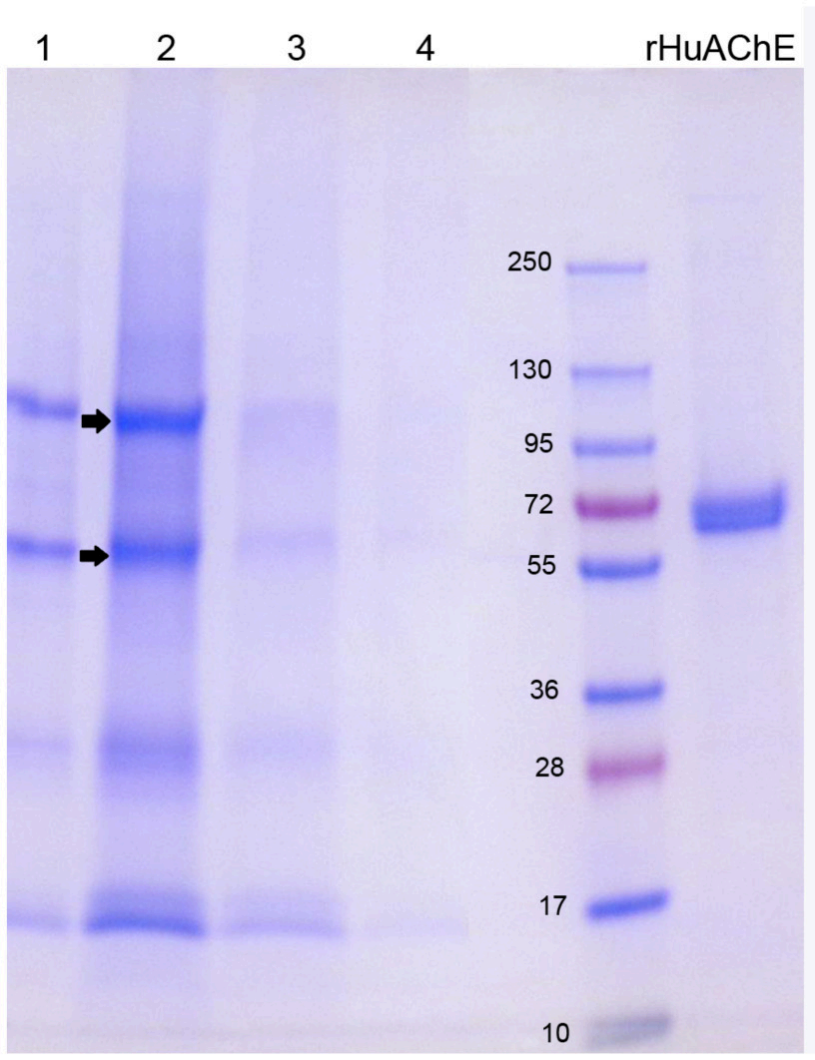
SDS gel of RBC AChE eluted from Hupresin with 1% TFA. 1 mL fractions were collected, dried, dissolved in SDS/DTT gel loading buffer and loaded on a 4–20% precast gel. Fractions each have about five protein bands. The arrows point to bands that were analyzed by mass spectrometry and found to include AChE. Control rHuAChE has a mass of 65 kDa.

The SDS gel in Figure 4 shows the proteins that eluted from 2 mL Hupresin with 1% TFA. Fraction 2 (in lane 2) has 4 or 5 bands, evidence that most red cell proteins had washed off Hupresin before the Hupresin column was eluted with 1% TFA. Proteomic analysis has identified 2,838 proteins in the human erythrocyte (D'Alessandro et al., 2017). The control sample, recombinant human AChE (rHuAChE), has a mass of 65 kDa. Reduced RBC AChE has a molecular weight of 75 kDa (Rosenberry and Scoggin, 1984). None of the TFA fractions have a 75 kDa band. The bands in lane 2 marked with arrows were cut out of the gel, digested with trypsin and analyzed by LC-MS/MS. Human AChE (accession P22303) was the most abundant protein identified in both bands. Each band also had other proteins, the most abundant being plasminogen P00747, retinal dehydrogenase P00352, actin P63261, spectrin P02549, and hemoglobin beta P68871. These proteins are known components of the human erythrocyte (D'Alessandro et al., 2017). It was concluded that RBC AChE bound to Hupresin and was recovered in 1% TFA.

## Discussion

### Cleaning and sanitation of hupresin with sodium hydroxide

It is common practice in industry to clean and sanitize chromatography media with sodium hydroxide. Solutions of sodium hydroxide remove proteins, nucleic acids, fat, and endotoxin. Treatment of chromatography media with sodium hydroxide inactivates virus, fungi, yeast, prions, and bacteria (GE-Healthcare, 2014). Affinity media are susceptible to loss of the ligand during exposure to the high pH of a 0.1 M NaOH solution. We tested the stability of Hupresin to seven repeated treatments with 0.1 M NaOH for 45 min at room temperature by measuring the binding capacity for BChE from human plasma and for AChE solubilized from human RBC. We found the binding capacity was unchanged. Furthermore, we noticed that Hupresin, which acquired a brown color after chromatography of no-ghost RBC AChE, was returned to its pristine white color after being washed with 0.1 M NaOH. It was concluded that Hupresin can be washed with 0.1 M NaOH without loss of binding capacity for BChE and AChE.

### Antibody beads vs. hupresin for purifying BChE from plasma

We have been using Hupresin for several years to purify BChE from frozen Cohn fraction IV-4. We use ion exchange chromatography on Q-ceramic media at pH 4.5 followed by affinity chromatography on Hupresin at pH 8. The BChE eluted from Hupresin has BChE activity and is 99% pure. Pure BChE has only two bands on Coomassie blue stained SDS gels, the BChE monomer at 85 kDa and the non-reducible dimer at 170 kDa, as seen in Figures 1, 2. These results are emphasized here to make clear that Hupresin is an excellent affinity gel for purifying human plasma BChE in a 2-step chromatography protocol. However, in a one-step chromatography protocol Hupresin yields 10–15% pure BChE. We found that immunopurification of BChE from plasma on immobilized monoclonal antibodies mAb2 or B2 18-5 yields purer BChE protein than a one-step affinity chromatography of plasma on Hupresin.

The disadvantage of immunopurification of BChE is that BChE cannot be released from the antibody complex without unfolding and denaturing the BChE protein. Denatured BChE is useful for studies whose aim is to detect exposure to nerve agents, where denatured BChE is digested with pepsin and analyzed by LC-MS/MS (Fidder et al., 2002; Sporty et al., 2010; Carter et al., 2013; Pantazides et al., 2014; Mathews et al., 2017).

Hupresin has the advantage that BChE is eluted with 0.1 M trimethylammonium bromide, a mild solvent that does not destroy BChE enzyme activity or subunit association. A second advantage is sample size. Hupresin affinity columns can be scaled up to process hundreds of liters of plasma or Cohn paste extract, whereas immobilized antibodies typically immunopurify BChE from <0.5 mL plasma (Sporty et al., 2010; Carter et al., 2013; Pantazides et al., 2014; Mathews et al., 2017).

### Hupresin for purifying RBC AChE

Hupresin can be used to purify RBC AChE for studies that require denatured AChE, but Hupresin is not a good choice for purifying native AChE because the binding is so tight that active AChE is recovered in very low yield. A good choice for purifying active AChE is the procainamide affinity gel. This gel was developed for purification of human plasma BChE (Lockridge et al., 2005), but it works even better for purifying human AChE. We routinely purify rHuAChE on procainamide affinity gels.

### No-ghost method for purification of RBC AChE

AChE is associated with the membrane in RBC. In the traditional method for purifying proteins from RBC membranes, red cells are lysed and washed extensively to remove hemoglobin, leaving a white pellet of red blood cell membranes called ghosts (Dodge et al., 1963). We found that the hemoglobin depletion step could be omitted for samples to be immunopurified and for samples to be chromatographed on Hupresin. When RBC are treated with 0.6% Triton X-100 in PBS plus 0.1% azide the membrane-bound AChE is solubilized. After removal of insoluble debris the solution is very red, owing to the released hemoglobin. However, the hemoglobin and essentially all the proteins released from the RBC do not stick to Hupresin (or to anti-AChE antibodies) providing a one-step method for highly enriching AChE from RBC. We named this method “no-ghost” to distinguish it from the traditional method of preparing red blood cell ghosts before extracting the AChE. Recovery of AChE from Hupresin (or antibodies) requires denaturing reagents such as 1% TFA which render the AChE inactive.

## Author contributions

SO and OL performed experiments. LS set up mass spectrometry methods and data searches. ED synthesized Hupresin. SO, ED, OT, LS, and OL wrote the paper.

### Conflict of interest statement

The authors declare that the research was conducted in the absence of any commercial or financial relationships that could be construed as a potential conflict of interest.

## Abbreviations

AChE: acetylcholinesterase (P22303)
BChE: butyrylcholinesterase (P06276)
DHB: 2,5-dihydroxybenzoic acid
PBS: phosphate buffered saline
LC-MS/MS: liquid chromatography tandem mass spectrometry
MW: molecular weight
RBC: red blood cell
TFA: trifluoroacetic acid
TMA: tetramethylammonium bromide.
